# Atorvastatin‐Associated Liver Injury: Outcome After Statin Rechallenge

**DOI:** 10.1111/fcp.70073

**Published:** 2026-02-15

**Authors:** Blandine Bertin, Valentine Lacotte, Jean‐Luc Cracowski, Anais Gaiffe, Jérôme Dumortier, Thierry Vial

**Affiliations:** ^1^ Pharmacovigilance Center, Hospital University Pharmacotoxicology Department Hospices Civils de Lyon Lyon France; ^2^ Pharmacovigilance Unit Grenoble Alpes University Hospital Grenoble France; ^3^ HP2 Laboratory, Inserm U1300 Université Grenoble Alpes Grenoble France; ^4^ Franche‐Comté Regional Pharmacovigilance and Drug Information Center Besançon University Hospital Besançon France; ^5^ Department of Digestive Diseases, Hospices Civils de Lyon, Edouard Herriot Hospital Claude Bernard Lyon 1 University Lyon France

**Keywords:** adverse drug reaction, drug‐induced liver injury, hydroxymethylglutaryl‐CoA, reductase inhibitors, recurrence, retreatment

## Abstract

**Background:**

Statin‐induced liver injury is frequent and usually not severe. The aim of the present study was to describe the safety of statin rechallenge after atorvastatin‐induced liver injury because it is poorly documented.

**Methods:**

Cases of liver injury involving atorvastatin were selected from the French pharmacovigilance database. Inclusion criteria were a documented atorvastatin or any other statin reintroduction and an available follow‐up of at least 2 weeks to define negative rechallenge.

**Results:**

Twenty‐six cases of atorvastatin liver injury with further statin reintroduction met our criteria. Median time to onset (TTO) of the first episode was 27 days (IQR: 4–43), with a cholestatic pattern in 11 (42.3%) cases, cytolytic in nine (34.6%), and mixed in six (23.1%); severity ranked Grade 2 in 11 (42.3%). Atorvastatin rechallenge was positive in 12 of 16 patients with the same dose (11 of 13) or a reduced dose (1 of 3), and the TTO was shorter (median 11 days). Rechallenge with an alternative statin was performed in 10 patients, of whom two experienced recurrence with rosuvastatin and simvastatin. No recurrence was observed after rechallenge of rosuvastatin in five, pravastatin in two, and simvastatin in one.

**Conclusion:**

Our study evidenced frequent recurrence of drug‐induced liver injury after atorvastatin rechallenge, whereas subsequent administration of a hydrophilic statin was well tolerated. By combining our data and published cases, we suggest that rosuvastatin or pravastatin carries the lowest risk of recurrence. Study limitations include a focus solely on atorvastatin, a retrospective design, and potential underreporting to the pharmacovigilance system.

**Abbreviations**
ADR: adverse drug reaction
ALP: alkaline phosphatase
ALT: alanine aminotransferase
ANSM: Agence Nationale de Sécurité des Médicaments et des produits de santé
DILI: drug‐induced liver injury
FPD: French pharmacovigilance database
HMG‐CoA reductase: hydroxymethylglutaryl‐CoA reductase
MedDRA: Medical Dictionary for Regulatory Activities
TTO: time to onset
ULN: upper limit of normal

## 1 Introduction

Since the discovery of the hydroxyméthylglutaryl‐CoA réductase (HMG‐CoA reductase) in 1973, several statins have been developed to reduce the cardiovascular risk associated with hypercholesterolemia [1]. Lovastatin was first marketed in 1987, followed by simvastatin (1988), pravastatin (1991), fluvastatin (1994), atorvastatin (1997), cerivastatin (1998, withdrawn due to excessive risk of rhabdomyolysis), and rosuvastatin (2003). Atorvastatin is actually the most widely prescribed statin worldwide including in France [2].

Muscle symptoms and liver enzyme abnormalities are the most frequent adverse effects associated with statins [3, 4]. The liver toxicity usually consists of mild‐to‐moderate and asymptomatic increases in serum transaminase levels that may resolve even with treatment continuation. The incidence of liver transaminase increases is estimated to range between 1% and 3% of patients with most statins [5, 6, 7], and an incidence of 0.7% has been reported with atorvastatin [8, 9].

Clinically significant drug‐induced liver injury (DILI) associated with statins is uncommon and may manifest in a variety of forms, including hepatocellular, cholestatic, or mixed lesions [10, 11]. In some instances, the liver injury may exhibit autoimmune characteristics [10]. The incidence of DILI associated with statins has been estimated from a multicenter US study, with the highest incidence reported for atorvastatin (1:10 000 prescriptions) [12]. Data derived from two distinct database studies also identified atorvastatin as the causal statin in 41% (30/73) and 34% (16/47) of statin‐associated liver injury cases [13, 14]. Data on the frequency of DILI with other statins are more heterogeneous between studies [12, 13, 14].

In patients diagnosed with statin‐induced acute liver injury, the decision to restart a statin has practical and ethical concerns but may be crucial in patients with high‐risk factors. In such instances, data supporting that there is no potential risk of developing severe liver injury after statin reintroduction is therefore mandatory. Unfortunately, the risk of recurrence of liver injury following the reintroduction of atorvastatin or another statin after atorvastatin‐induced DILI is poorly described in the literature, and cases associated with both a positive and negative rechallenge were reported [14, 15].

Our primary objective is to describe the characteristics of atorvastatin‐induced DILI and their outcome after rechallenge with atorvastatin or another statin.

## 2 Methods

### 2.1 Study Design

Cases were selected from the French pharmacovigilance database (FPD), which contains anonymized data on spontaneous adverse drug reactions (ADRs) reported in France since 1985. ADRs were coded according to the Medical Dictionary for Regulatory Activities (MedDRA) [16]. A drug is considered suspect if it is thought to have caused the ADR, concomitant if not, or interacting if the ADR is compatible with a drug–drug interaction. An ADR is considered serious if it leads to death, is life‐threatening, results in a permanent disability/incapacity, causes hospitalization or the prolongation of a hospitalization, or is medically relevant. The study was approved by the 31 French regional centers of pharmacovigilance and the ANSM (Agence Nationale de Sécurité des Médicaments et des produits de santé). The data that support the findings of this study are available on request from the corresponding author and after agreement from ANSM. The study was designed and reported in accordance with the STROBE (Strengthening the Reporting of Observational Studies in Epidemiology) guidelines [17]. Because of the retrospective and noninterventional design of this study, the approval of the local Ethics Committee was not necessary. The study was not registered in a public registry.

### 2.2 Cases Selection

Reports involving atorvastatin coded as suspect or interacting drug and associated with the MedDRA HLGT “Hepatobiliary investigations” or “Hepatic and hepatobiliary disorders” were extracted from the FPD. The study was limited to the period March 21, 1997, the first marketing authorization date for atorvastatin in France, to August 31, 2023. All cases and their narratives were individually scrutinized and selected according to predefined inclusion and exclusion criteria. Case selection was done independently by two reviewers, with a third person to settle in case of disagreements.

Inclusion criteria were a documented rechallenge with any statin after recovery of atorvastatin‐associated DILI and a documented follow‐up of at least 2 weeks after reintroduction. To ensure the clinical significance of the liver injury, we used the clinical chemistry criteria for DILI as proposed by Aithal et al. with at least a two‐fold and a five‐fold increase in alkaline phosphatase (ALP) and alanine aminotransferase (ALT), respectively [18].

Exclusion criteria were significant pre‐existing liver damage, another more likely drug‐related cause or nondrug cause, associated signs of rhabdomyolysis, lack of biological recovery despite atorvastatin withdrawal, unknown outcome or insufficient follow‐up time after statin reintroduction, noncompliance with the predefined biological criteria, and poorly documented cases.

### 2.3 Handling of Data

Data on age, sex, medical history, drug exposure (start and stop dates of the drug or treatment duration), adverse effect (onset date or time to onset, outcome, date of recovery or event duration, and severity), and the complete narrative were extracted. Dates coded in the database were used to calculate time periods (e.g., duration of the treatment, time to onset of the event, and duration of liver abnormalities). When missing, they were extracted from the case narrative. When incomplete, we rounded them. The narrative also helped to identify any other alternative cause and detailed results of additional investigations.

For each case, liver test values were converted into multiples of the upper limit of normal (ULN). If the normal values were not specified, we used the normal values from the Tietz Clinical Guide of Laboratory Tests [19]. The pattern of DILI (cholestatic, mixed, and cytolytic) was classified according to the *R* value with *R* = ALT/ALP [18].

We also used the classification of liver injuries into the four grades of severity established by Aithal et al.

The causal relationship between liver injury and atorvastatin exposure was assessed using the method of international consensus meeting [20].

Positive rechallenge was retained if the liver injury recurred after any statin readministration, whereas the first episode had resolved, irrespective of the time to onset of recurrence. According to a timeframe based on literature data, a negative rechallenge was defined by the lack of recurrence of the liver injury after at least 2 weeks of follow‐up after restarting a statin [21, 22].

### 2.4 Literature Review

A literature review was performed to identify relevant case reports pertaining to statin rechallenge after atorvastatin‐induced DILI. PubMed/MEDLINE was searched without time restriction on September 25, 2024, using the search strategy detailed in Appendix A. General reviews and the reference lists of all the originally retrieved articles were also screened to further identify additional relevant articles. Non‐English or French studies and studies with insufficient data were excluded.

## 3 Results

### 3.1 Case Series Description

**FIGURE 1:**
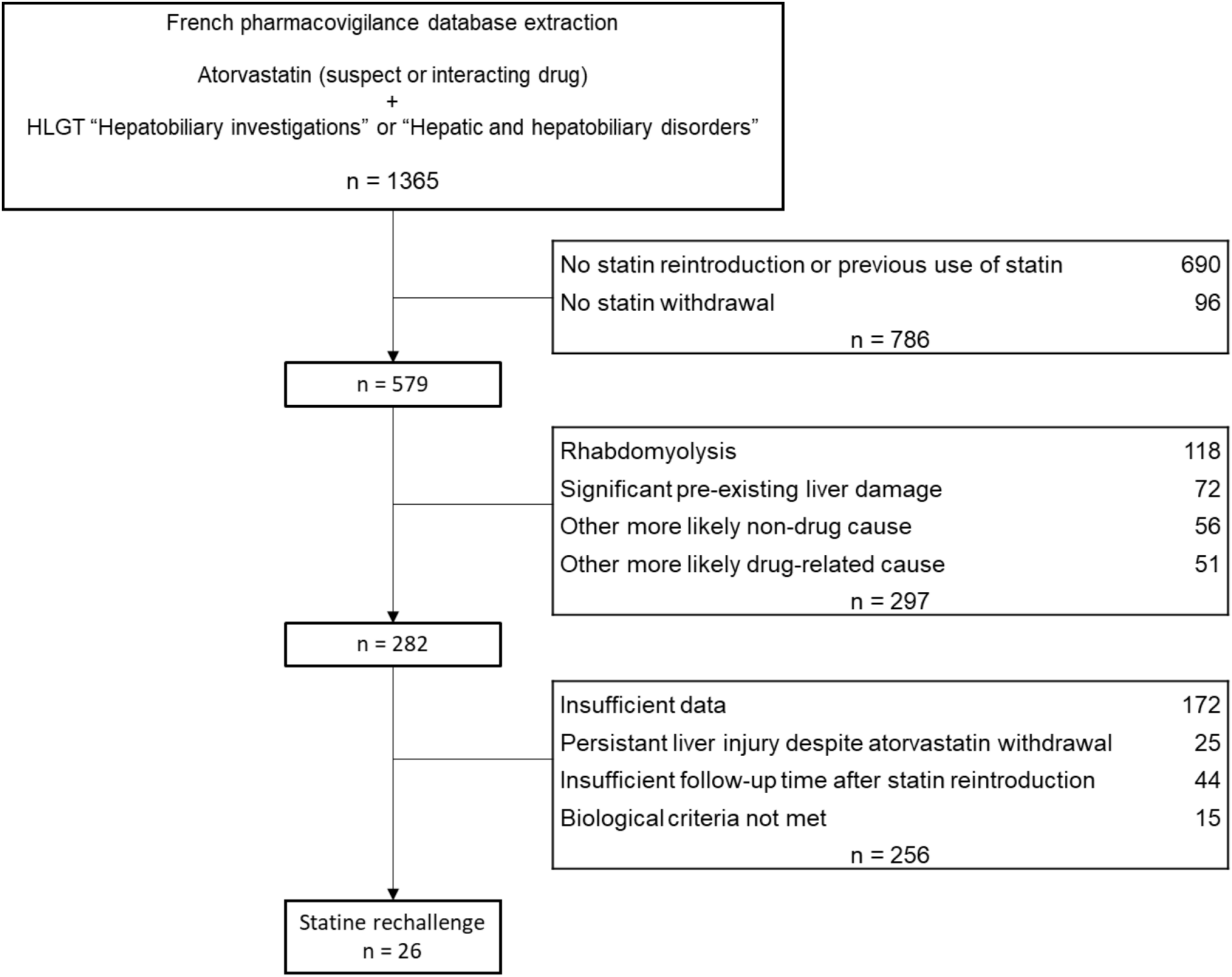
Flow chart.

Of the 1365 cases of atorvastatin‐associated liver injury extracted from the FPD, 26 fulfilled our criteria and were included in the final analysis (Figure 1).

The main characteristic of the 26 cases is indicated in Table 1. A current or recent history of alcohol abuse was reported in two patients. Among the 26 cases, a single case occurred after more than 4 months of treatment and was associated with the recurrence of liver injury after atorvastatin rechallenge. The pattern of the liver injury was mostly cholestatic (42.3%). Three patients with a cytolytic pattern also had a bilirubin concentration above two‐fold the ULN. Slightly more patients were classified with mild (Grade 1) DILI severity index, and 10 had clinically symptomatic liver injury.

**TABLE 1:** Main characteristics of atorvastatin‐induced liver injury (*n* = 26).

Female, *n* (%)	14 (53.8)
Age (years), median (IQR)	75 (64–81)
Time to onset (days), median (IQR) (*n* = 22)	27 (4–43)
Daily dose, median (IQR) (*n* = 23)	40 mg (30–80)
Type of liver injury, *n* (%)	
Cholestatic^a^	11 (42.3)
Cytolytic^b^	9 (34.6)
Mixed pattern	6 (23.1)
Grade of the liver injury, *n* (%)	
Grade 1 (mild)	15 (57.7)
Grade 2 (moderate)	11 (42.3)
Duration of liver injury (days), median (IQR) (*n* = 21)	22 (15–41)

^a^ Median value of PAL was six‐fold the ULN (IQR 4–8).

^b^ Median value of ALT was nine‐fold the ULN (IQR 8–19).

A liver biopsy was available for three patients. An inflammatory portal or periportal pattern suggesting DILI or an autoimmune pattern was evidenced in two patients, whereas features of DILI associated with a subcapsular hematoma were found in the third patient. Liver autoantibody test results were available for five patients. Two tested negative, and three were positive for at least one autoantibody including antinuclear antibodies in three, antimitochondrial antibodies in two, and anti‐double‐stranded DNA in one. The abdominal ultrasound performed in 15 patients was normal in nine and not contributive in one. Abnormalities identified in the five remaining patients consisted of minimal bile duct dilatation without biliary obstruction, isolated dilatation of the subhepatic veins, homogeneous hepatomegaly with uncomplicated vesicular lithiasis, uncomplicated vesicular sludge without bile duct dilatation, and signs of angiocholitis. This last case was retained as the patient further experienced positive atorvastatin rechallenge, whereas the liver injury had completely resolved after a cholecystectomy. Viral hepatitis serologies were available for 14 patients and were negative for hepatitis A in nine, hepatitis B in 11, hepatitis C in 12, and hepatitis E in five (detailed results not provided in one).

Of these 26 patients, 16 were later rechallenged with atorvastatin, and 10 received another statin after complete biological recovery.

Among the 16 patients who restarted atorvastatin, 13 received the same dose, and three received a reduced dose. The median follow‐up duration after rechallenge was 40 days (IQR 29–53), regardless of the outcome. DILI recurred in 11 of the 13 patients who received the same atorvastatin dose and in one of the three patients who received a reduced dose. The median time between atorvastatin reintroduction and reappearance of the DILI was 11 days (IQR: 6–14; data available in 12 patients) with 2 days as the shortest time in two patients. Although this time to onset was shorter than the first episode, the difference was not significant. The second episode of liver injury was more severe in only one patient who developed a Grade 2 cholestatic injury within 13 days after rechallenge of the same atorvastatin dose and with complete recovery after definitive drug discontinuation. The biological pattern of the second episode was similar in five patients, whereas it changed from a cholestatic to a cytolytic or a mixed pattern in two, from a mixed to a cholestatic pattern in two, and from a cytolytic to a cholestatic pattern in three. The liver injury relapsed after the resumption of atorvastatin in the single patient from this group in whom autoimmunity tests were positive. Medical history also revealed that one patient with positive atorvastatin rechallenge previously received pravastatin uneventfully.

Ten patients were switched to another statin that consisted of rosuvastatin in six, pravastatin in two, and simvastatin in two. No recurrence of the liver injury was observed in eight patients after a median follow‐up of 33 days [IQR: 25–55], including one who previously and subsequently tolerated simvastatin. The last two patients experienced relapse of the DILI within 1 day and 88 days after the switch to rosuvastatin and simvastatin, respectively. Unfortunately, no liver tests were available before this date in this last patient. Compared to the initial episode, the grade of the liver injury was similar in one patient and less severe in the second patient. Of the two patients with previous positive autoimmunity tests for both antinuclear and antimitochondrial antibodies, one relapsed within 3 months of simvastatin treatment, while the other received rosuvastatin uneventfully.

### 3.2 Literature Review

Our search strategy identified 11 case reports or case series involving 14 patients (nine females; median age 68 years [IQR: 59–71]) who experienced atorvastatin‐induced liver injury followed by a subsequent rechallenge with atorvastatin or another statin [14, 15, 23, 24, 25, 26, 27, 28, 29, 30, 31]. The median daily dose of atorvastatin was 40 mg (IQR 20–80). The time to onset of DILI documented in only two patients was 60 and 90 days, and the pattern of the liver injury was cytolytic in seven patients, cholestatic in two, and mixed in two (unknown in three). Of the seven patients tested for autoimmunity, two were positive for antinuclear antibodies, anti‐PDC (M2), or anti‐smooth muscle antibodies. The median time to recovery in eight patients with available data was 30 days (IQR 14–31). According to Aithal et al. criteria, the liver injury was Grade 1 in eight patients (57%) and two in 6 (43%). Of these 14 patients, seven restarted atorvastatin, including one at a reduced dose. All seven patients experienced a recurrence of the liver injury with the same pattern, and one developed a more severe cytolytic liver injury. A switch to another statin was performed in 10 patients, including three patients who previously had positive atorvastatin rechallenges. None experienced recurrence of the liver injury after the use of pravastatin in four, simvastatin in three, lovastatin in two, and rosuvastatin in one.

**FIGURE 2:**
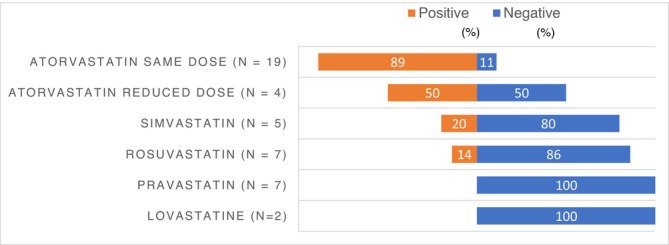
Distribution of positive and negative statin rechallenges, combining cases from our study and the literature.

Combining our data with the published literature clearly confirms a high risk of recurrence after atorvastatin readministration, even at lower doses, with an overall recurrence rate of 83% (Figure 2). By contrast, liver injury did not recur in all eight patients further receiving pravastatin, in six of seven in those receiving rosuvastatin, and in four of five in those receiving simvastatin.

## 4 Discussion

In our case series, both the TTO of atorvastatin‐induced liver injury and the duration of the reaction after its discontinuation were suggestive of a DILI as proposed at an international consensus meeting on drug‐induced liver disorders [20]. Most of our patients developed a cholestatic injury comparable to that previously published with atorvastatin [14]. The biological or histological features of auto‐immune liver injury evidenced in six patients were also reminiscent of other similar isolated cases reported with atorvastatin [25, 32]. Although several of our patients who presented a cytolytic pattern showed a dramatic increase in liver transaminases, none exhibited clinical or biological signs of severe liver injury.

The high recurrence rate of liver injury after atorvastatin reintroduction in our patients is lower but in accordance with the systematic recurrence observed in published case reports [14, 15, 23, 29, 30]. Such a recurrence rate and the shorter TTO of liver injury after atorvastatin rechallenge are definitely suggestive of DILI. Reassuringly, the second episode was more severe in only one patient rechallenged with atorvastatin and remained self‐limited in all others. Conversely, the administration of another statin was well‐tolerated in most of our patients with no case of recurrence after pravastatin readministration and only two instances of positive rechallenge with rosuvastatin and simvastatin, and a benign outcome in both patients. Data from the literature indicated less contrasting results with a negative rechallenge of another statin in all instances. Overall, and even if limited to very few cases, we still identified instances of negative rechallenge with atorvastatin as well as positive rechallenge with another statin that contrasted with the current literature.

The safety of statin rechallenge in patients who initially exhibited features of autoimmune liver injury is particularly challenging. Combining data from our study and the literature, five patients initially had biological or histological signs suggestive of autoimmune liver injury and consistent with the published data identifying atorvastatin as the statin most frequently mimicking idiopathic autoimmune liver injury [32]. Of these patients, two experienced recurrence of the liver injury when atorvastatin was resumed, one had a recurrence with simvastatin, and the other two uneventfully received rosuvastatin or pravastatin. As suggested by Björnsson et al. [32] simvastatin was the second statin most frequently associated with autoimmune DILI. Accordingly, we suggest preferably not using this statin in such patients.

The mechanism of atorvastatin‐induced liver injury is unclear. It may involve CYP450‐dependent metabolism, leading to the generation of reactive oxygen species and mitochondrial dysfunction [6, 33, 34]. Clinically significant liver damage caused by statins may also result from autoimmune, idiosyncratic, and immunoallergic mechanisms. Given its lipophilic nature and extensive metabolism by CYP3A4, the hepatotoxicity of atorvastatin would be significantly enhanced [35]. Some authors have suggested that high daily doses of drugs extensively metabolized by hepatic CYP enzymes, such as atorvastatin, may increase the risk of idiosyncratic DILI [36, 37]. The mean daily dose of 40 mg observed both in our cohort and in the literature lends support to this assumption.

Conversely, hydrophilic statins that are not metabolised by the CYP 3A4 metabolism are hypothesised to have a lower risk of liver injury. Our data combined with the published reports is consistent with this approach as pravastatin, a hydrophilic drug with minimal CYP‐dependent metabolism [38], was not associated with liver injury recurrence. Rosuvastatin, another poorly metabolized hydrophilic drug, was also tolerated in most patients. Data with simvastatin are more contrasted. Given its similarity with atorvastatin (lipophilic nature and metabolism by the same cytochrome) [5], it seems reasonable to expect a recurrence.

Given the small number of cases previously published, the main strength of our study is the significant number of cases selected on the basis of stringent biological criteria, appropriate follow‐up duration after rechallenge, and collection of the data at a national level. Our study confirms the high risk of recurrence after atorvastatin rechallenge, even at lower doses, and highlights the possibility of switching to an alternative statin even after a significant episode of atorvastatin liver injury. Moreover, no worsening of the liver injury was observed in the rare instances of positive rechallenge with a hydrophilic statin. Most of the cases of drug rechallenge were also prospectively documented through follow‐up of the initial case and independently of the seriousness of the first episode. This might have limited a possible reporting bias as suggested from the previous literature that systematically reported positive rechallenge after restarting atorvastatin and negative rechallenge with other statins [14, 15, 23, 24, 25, 26, 27, 28, 29, 30, 31].

The main limitation of our study is the restriction to cases of atorvastatin liver injury that do not allow general assessment of possible hepatotoxic cross‐reactions between statins. This decision was made on the basis that atorvastatin is the most commonly used [14] and the most frequently associated with medically significant DILI [13, 14]. The second limitation is the retrospective nature of reporting with the known biases related to this type of study. In particular, a number of cases were not fully documented as regards possible other causes or lacked essential information that led us to exclude a significant number of reports. Some cases may also not have been correctly captured due to heterogeneity or errors in coding procedures. The third limitation is the well‐known underreporting to the pharmacovigilance system as it is estimated that barely 5% of all serious ADRs are reported [39]. Finally, a selective reporting bias should also be considered with serious cases more likely to be reported. In addition, the study was not registered in a public registry, which should be acknowledged as a limitation.

## 5 Conclusion

Our study evidenced frequent recurrence of liver injury after atorvastatin rechallenge, even with a reduced dose. Data on the safety of subsequent rechallenge with simvastatin is still limited, and we advise against its use based on pharmacokinetic similarities with atorvastatin. By contrast, and combining our data with the previously published cases, we suggest that the hydrophilic pravastatin or rosuvastatin carries the lowest risk of recurrence.

## Author Contributions

All authors contributed to the study conception and design. Data collection was performed by B.B., J.‐L.C., A.G., and T.V. Material preparation and analysis were performed by B.B., V.L., and T.V. The first draft of the manuscript was written by B.B. and T.V., and all authors commented on previous versions of the manuscript. All authors read and approved the final manuscript.

## Funding

The authors received no specific funding for this work.

## Ethics Statement

Because of the retrospective and noninterventional design of this study, the approval of the local Ethics Committee was not necessary.

## Consent

The authors have nothing to report.

## Conflicts of Interest

The authors declare no conflicts of interest.

## Data Availability

The data that support the findings of this study are available on request from the corresponding author and after agreement from ANSM. The data are not publicly available due to ethical restrictions and to preserve individuals' privacy under the European General Data Protection Regulation.

## 1

(“Chemical and Drug Induced Liver Injury”[Mesh] OR “Liver Diseases”[Mesh] OR hepat* OR liver OR Liver Injury OR hepatitis OR Toxic Hepatitis OR Toxic Hepatitides OR Drug‐Induced Liver Diseases OR Drug‐Induced Liver Injury OR Drug‐Induced Liver Injuries OR Drug‐Induced Liver Disease OR Drug Induced Liver Diseases OR Drug Induced Liver Injury OR Drug Induced Liver Injuries OR Drug Induced Liver Disease OR Drug‐Induced Hepatitides OR Drug‐Induced Hepatitis OR Drug Induced Hepatitides OR Drug Induced Hepatitis)

AND

(“Hydroxymethylglutaryl‐CoA Reductase Inhibitors”[Mesh] OR hydroxymethylglutaryl‐coa reductase inhibitors OR hydroxymethylglutaryl‐coa reductase inhibitor OR HMG CoA Reductase Inhibitor OR hmg coa reductase inhibitors OR HMG‐CoA Reductase Inhibitor OR hmg‐coa reductase inhibitors OR statins OR Statin OR Statin*)

AND

(“Retreatment”[Mesh] OR rechallenge OR reintroduction OR re‐introduction OR resumption)
